# Metabolic Profiling by UPLC–Orbitrap–MS/MS of Liver from C57BL/6 Mice with DSS-Induced Inflammatory Bowel Disease

**DOI:** 10.1155/2020/6020247

**Published:** 2020-09-23

**Authors:** Zhongquan Xin, Zhenya Zhai, Hongrong Long, Fan Zhang, Xiaojun Ni, Jinping Deng, Lunzhao Yi, Baichuan Deng

**Affiliations:** ^1^Maoming Branch, Guangdong Laboratory for Lingnan Modern Agriculture, Guangdong Provincial Key Laboratory of Animal Nutrition Control, National Engineering Research Center for Breeding Swine Industry, College of Animal Science, South China Agricultural University, Guangzhou, China; ^2^Institute of Biological Resource, Jiangxi Academy of Sciences, Nanchang 330029, China; ^3^Yunnan Animal Science and Veterinary Institute, Jindian, Panlong County, Kunming City, Yunnan Province, China; ^4^Yunnan Food Safety Research Institute, Kunming University of Science and Technology, Kunming 650500, China

## Abstract

Liver disorder often occurs in patients with inflammatory bowel disease (IBD); however, the changes in IBD-induced liver disorder at the intrinsic molecular level (chiefly metabolites) and therapeutic targets are still poorly characterized. First, a refined and translationally relevant model of DSS chronic colitis in C57BL/6 mice was established, and cecropin A and antibiotics were used as interventions. We found that the levels of tumor necrosis factor (TNF)-*α*, interleukin (IL)-1*β*, and IL-6 in the liver tissues of mice were highly increased in the context of DSS treatment but were lowered by cecropin A and antibiotics. Subsequently, an untargeted metabolomics analysis was performed by UPLC–Orbitrap–MS/MS to reveal the metabolic profile and attempt to find the potential therapeutic targets of the liver disorders that occur in IBD. Notably, 133 metabolites were identified by an integrated database. Metabolism network and pathway analyses demonstrated that the metabolic disturbance of the liver in IBD mice was mainly enriched in bile acid metabolism, arachidonic acid metabolism, amino acid metabolism, and steroid hormone biosynthesis, while those disturbances were regulated or reversed through cecropin A and antibiotic treatment. Furthermore, the top 20 metabolites, such as glutathione, maltose, arachidonic acid, and thiamine, were screened as biomarkers via one-way analysis of variance (one-way ANOVA, *p* < 0.05) coupled with variable importance for project values (VIP >1) of orthogonal partial least-squares discriminant analysis (OPLS-DA), which could be upregulated or downregulated with the cecropin A and antibiotics treatment. Spearman correlation analysis showed that the majority of the biomarkers have a significant correlation with cytokines (TNF-*α*, IL-1*β*, IL-6, and IL-10), indicating that those biomarkers may act as potential targets to interact directly or indirectly with cecropin A and antibiotics to affect liver inflammation. Collectively, our results extend the understanding of the molecular alteration of liver disorders occurring in IBD and offer an opportunity for discovering potential therapeutic targets in the IBD process.

## 1. Introduction

Inflammatory bowel disease (IBD) is a complex chronic disease of the gastrointestinal tract, including Crohn's disease (CD) and ulcerative colitis (UC) [1]. As a systemic disorder, IBD has been associated with many extraintestinal manifestations (EIMs) involving almost all organ systems, including the musculoskeletal, dermatologic, renal, hepatobiliary, pulmonary, and ocular systems [2]. The liver is the main metabolic organ that could interact with the intestinal tract directly through the hepatic hilum and/or bile secretion system; therefore, liver disorder is frequently observed in IBD and along with various hepatobiliary diseases, including fatty liver, autoimmune hepatitis, and cirrhosis [3]. It has been estimated that approximately 5% of patients with IBD develop serious liver diseases [4].

The invasion of pernicious bacteria such as *Escherichia coli (E. coli)* and *Salmonella* and/or the destruction of intestinal microbial structure is the main trigger of IBD [5]. Recent studies have found that intestinal metabolome disorders in patients with IBD are characterized by metabolic disorders of short-chain fatty acids, bile acids, and tryptophan [6], which are often correlated with liver metabolism [7–9]. It has been well accepted that antibiotics are commonly used to relieve IBD through the elimination of harmful bacteria and reduction of inflammation [10, 11]. Moreover, we also found that an antimicrobial peptide (cecropin A) can alleviate IBD [12]. Despite scientific advances in pathology studies on liver disorders associated with IBD, its metabolic and potential therapeutic targets are still obscure.

There are numerous ways (e.g., RT-qPCR and ELISA) to detect the liver response to exogenous stimuli; however, these methods only partially illustrate the changes in liver, and are thus limited and not comprehensive to evaluate the liver response to stimuli. Metabolomics is a popular technology and powerful high-throughput platform that can identify and quantify metabolites in organisms and find the relationship between metabolites and physiological/pathological changes [13, 14]. Ultrahigh-performance liquid chromatography coupled with high-resolution mass spectrometry (UPLC-HRMS) is one of the most efficient and robust methods of generating metabolite profiles for biological samples because of its high throughput, resolution, and sensitivity [15, 16]. To further understand the biological basis of the responses of the liver, it is necessary to explore the metabolic biology of IBD mice induced by DSS.

In the present study, an IBD model was established in C57BL/6 mice to induce liver inflammation, and cecropin A and antibiotics were used as interventions. ELISA was used to evaluate the inflammation of the liver based on the concentration of cytokines (TNF-*α*, IL-1*β*, and IL-6). The control group, the DSS group, the CA group (DSS + cecropin A treatment), and the GA group (DSS + antibiotics treatment) were used for untargeted metabolic analysis performed on a UPLC-Orbitrap-MS/MS. We aimed to reveal the metabolic profile of the liver in IBD and acquire biomarkers and potential therapeutic targets by metabolic pathway analysis.

## 2. Methods

### 2.1. Animals

Thirty-six C57BL/6 mice (7 ~ 8 weeks old) were kept in a mouse room without specific pathogens with a relative humidity of 45~60% and a 12 h/day lighting cycle (7 : 00 AM~7 : 00 PM for light) at 25 ± 1°C and divided into four groups (*n* = 9 mice per group). The refined and translationally relevant model of DSS chronic colitis in C57BL/6 mice was established by giving water containing 2.5% DSS for 5 days. After that, mice were intraperitoneally injected with or without cecropin A (15 mg/kg) or gentamicin (5 mg/kg) for 5 days. All mice were weighed every day and permitted ad libitum access to feed and water. All mice were sacrificed with CO2 inhalation (1 L/min, 5 min) followed by cervical dislocation to ensure death on the eighth day. The colon and liver of each mouse were collected for further processing.

The experimental design and procedures in this study were reviewed and approved by the Animal Care and Use Committee of the Institute of Subtropical Agriculture, Chinese Academy of Science (No.ISA-2018-035).

### 2.2. Chemicals and Reagents

Acetonitrile (LC-MS grade) and methanol (LC-MS grade) were purchased from Merck. Formic acid (LC-MS grade), gentamicin and dextran sulfate sodium (DSS, Mw 36,000~50,000) were purchased from J&K Scientific Limited. Cecropin A was synthesized by a peptide company (DgPeptides Co., Ltd., Hangzhou, China). Ultra-pure water was prepared from the ELGA system (RIGHTLEDER International Holding Group Limited).

### 2.3. Histopathological Examination

Tissue histological analysis was performed with hematoxylin and eosin (H&E). Fragments of the liver and colon were fixed in 4% neutral polyformaldehyde fixative, followed by dehydration, embedding in paraffin, cutting into 5-*μ*m slices, deparaffinization, hydration and staining. Thereafter, all tissue sections were examined by microscopy.

### 2.4. Elisa

To verify the liver disorder caused by IBD and the recovery effect of cecropin A and gentamicin, the levels of the cytokines TNF-*α*, IL-1*β*, IL-6, and IL-10 in the liver were detected by enzyme-linked immunosorbent assay (ELISA). Briefly, the liver tissues were weighed and homogenized in cold saline containing 0.1 mM phenylmethyl sulfonyl fluoride (PMSF), and the levels of cytokines were measured by commercial kits following the protocols (CUSABIO, Wuhan, China). The total protein was adjusted by the BCA assay kit (Thermo, Waltham, MA, USA).

### 2.5. Metabolite Extraction and Quality Control Sample

All of the tested liver samples were cut on dry ice and weighed. Liver tissue (~40 mg) was homogenized with a freeze tissue grinder, and homogenate (200 *μ*L) was moved to 1.5-mL EP tubes and mixed with 800 *μ*L of methanol/acetonitrile (1 : 1, v/v). The samples were vortexed for 30 s and centrifuged at 14,500 rpm for 15 min at 4°C. The supernatant was evaporated to dryness using nitrogen blowing followed by reconstitution with 100 *μ*L of acetonitrile/water (1 : 1, v/v). Finally, the solution was filtered with a 0.22-*μ*m membrane and used for UPLC-HRMS analysis.

The quality control (QC) sample was prepared by mixing equal volumes of different individual liver samples. A QC sample was inserted into every five samples during UPLC-HRMS analysis. The reproducibility and reliability of the method were assessed by the coefficients of variation (CV%) of metabolites from QC samples.

### 2.6. HRMS Analysis

The UPLC system was combined with a Q-Orbitrap mass spectrometer (Thermo Fisher Scientific, Q-Exactive Focus). A total of 2 *μ*L of sample solution was separated using a C18 column at 35°C with a linear gradient elution with 0.2% (v/v) formic acid solution (eluent A) and acetonitrile (eluent B) at a flow rate of 0.2 mL/min. A total 20-minute gradient program was set as follows: 0–8 min, 5–75% B; 8–11 min, 75–90% B; 11−15 min, 90−95% B, equilibration time of 5 min at 5% B. Xcalibur (version 3.0) was utilized for instrument control, data acquisition, and data analysis.

The MS analyses were conducted using electrospray ionization (ESI) modes and a full MS (m/z 70 to 1000) scan in positive ionization at a resolution of 35000. The ion spray voltage and heater temperature were kept at 3.5 kV and 300°C. MS/MS data were acquired using a data-dependent ms2 scan (dd-ms2) at a resolution of 17,000, and high collision-induced dissociation voltage (HCD) was set as a normalization stepped mode of 10, 30, 50.

### 2.7. Data Preprocessing and Metabolite Identification

Analytical instruments usually do not supply clean and visualized information on metabolites. Raw data must be processed to produce a workable data matrix through series preprocessing. These experiments were performed on the Compound Discoverer 2.1 (CD, Thermo Fisher Scientific) data analysis tool, which is flexible and able to automate complete preprocessing according to presupposed parameters. The CD has integrated various tools to identify small molecule metabolites, including search mzCloud (online spectral library >2 million spectra), ChemSpider (chemical structure database with >500 data sources, 58 million structures), mzVault (local spectral libraries), and Masslist (local databases). Data preprocessing and metabolite identification were completed by one-stop.

### 2.8. Statistical Analysis

Data in the current study were analyzed by one-way ANOVA using GraphPad Prism 6 (GraphPad Software LLC, San Diego, CA, USA) and SPSS 22.0 (SPSS Inc., Chicago, IL, USA) software, and all the data are shown as the mean ± standard error of the mean (SEM). Principal component analysis (PCA) and hierarchical cluster analysis (HCA) coupled with orthogonal partial least-squares discriminant analysis (OPLS-DA) were performed to investigate the data of liver metabolites, and biomarkers were selected by S-plot of OPLS-DA. The methods mentioned above were performed on SIMCA-14.1, R program, and Metaboanalyst 4.0 (http://www.metaboanalyst.ca). The values at *P* < 0.05 (∗), *P* < 0.01 (∗∗), and *P* < 0.001 (∗∗∗) were considered statistically significant.

## 3. Results

### 3.1. Evaluation of DSS-Induced IBD Mice and the Therapeutic Effects of Cecropin a and Gentamicin

**Figure 1:**
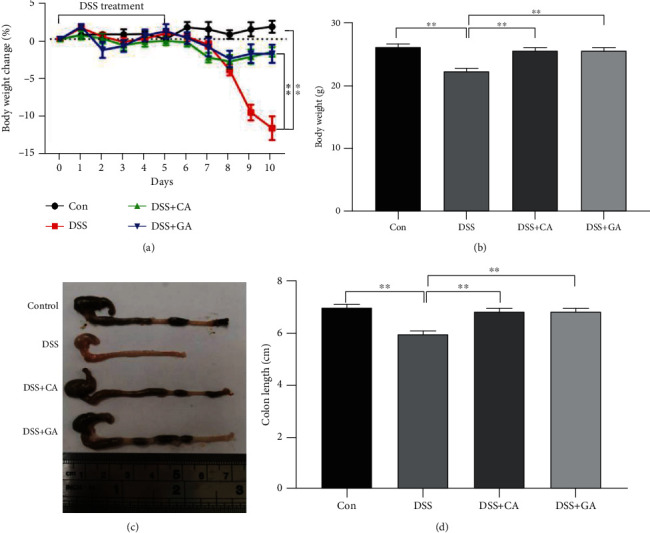
The effect of cecropin A and antibiotic treatment in 2.5% DSS-treated mice on body weight (a and b) and colon length (c and d). The data were analyzed by one-way ANOVA (*n* = 9) and are shown as the mean ± SEM with ∗*P* < 0.05, ∗∗*P* < 0.01, ∗∗∗*P* < 0.001 versus DSS.

The body weight and colon length of mice in the DSS group were both substantially reduced in comparison to those in the control group (*P* < 0.01, Figures 1(a)–1(d)). Besides In addition, the colon became less noticeable or even disappeared in the context of DSS treatment (Fig. S1a and b). These aforementioned findings vividly demonstrated that the refined and translationally relevant model of DSS chronic colitis in C57BL/6 mice was successfully established. Intriguingly, CA and GA both alleviated IBD symptoms, as evidenced by the increased body weight and colon length of IBD mice (*P* < 0.01, Figures 1(a)–1(d)).

### 3.2. Cecropin a and Gentamicin Both Attenuate DSS-Induced Liver Inflammation in Mice

**Figure 2:**
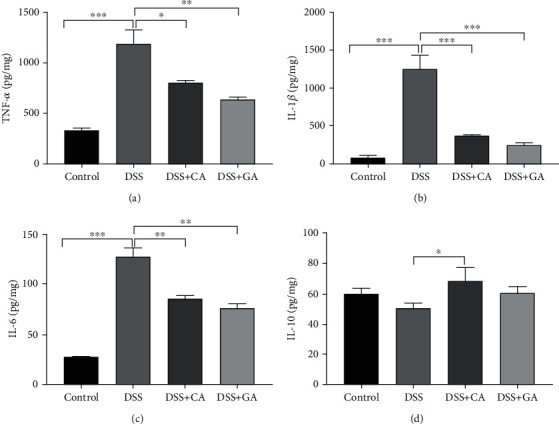
Local liver cytokine production in IBD mice and the effect of cecropin A and antibiotic treatment in DSS-treated mice. Protein concentrations of TNF-*α*, IL-1*β*, IL-6, and IL-10 per milligram of liver tissue by ELISA in the DSS group (*n* = 9), control group (*n* = 9), cecropin A group (CA, *n* = 9), and gentamicin group (GA, *n* = 9). The data were analyzed by one-way ANOVA and presented as the mean ± SEM with ∗*P* < 0.05, ∗∗*P* < 0.01, ∗∗∗*P* < 0.001 versus DSS.

Based on the H&E staining analysis, the liver morphology of mice in the DSS group showed hepatic cell swelling, cytoplasmic loosening, reticular structure and large numbers of apoptotic hepatocytes surrounding the vein (Fig. S1c and d), indicating the possible development of liver inflammation during IBD progression. ELISA was performed to detect the levels of inflammatory cytokines in livers, and DSS treatment was found to significantly increase TNF-*α*, IL-1*β*, and IL-6 levels (*P* < 0.001, Figure 2(a)–2(c)), while the levels of those proinflammatory cytokines were highly decreased after CA and GA treatment (*P* < 0.05, *P* < 0.01 or *P* < 0.001, Figures 2(a)–2(c)). Additionally, although DSS had little effect on its production, CA improved the level of IL-10 (*P* < 0.05, Figure 2(d)).

### 3.3. Evaluation of Analytical Performance

**Figure 3:**
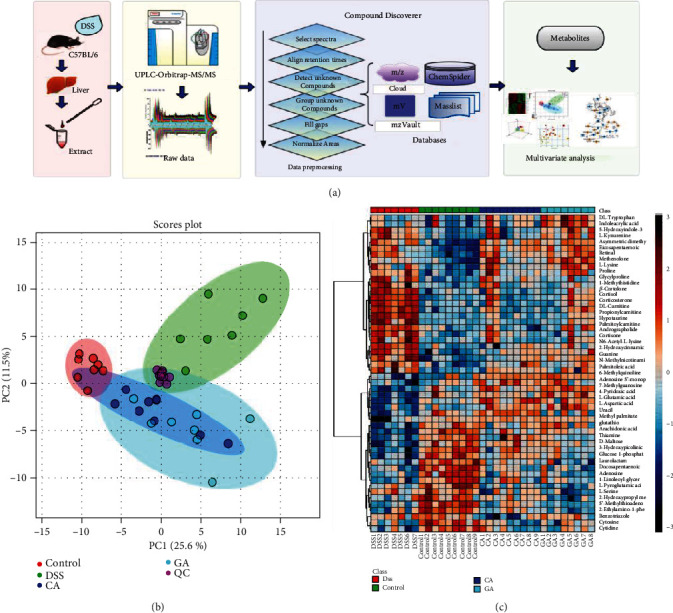
Metabolomics analysis flowchart and multivariate statistical analysis. (a) The study process diagram includes sample preprocessing, instrumental analysis, data preprocessing and qualitative analysis, multivariate screening and differential metabolite screening. (b) PCA of the DSS group, control group, cecropin A group (CA), gentamicin group (GA), and QC. (c) Heatmap analysis of the metabolites (*P* < 0.05) based on one-way ANOVA.

We conducted PCA on all samples, and the QC samples were tightly clustered in the middle of all samples in the PCA score plot, as shown in Figure 3(b). The coefficients of variation (CV %) of 133 metabolites from QC samples ranged from 2.74% to 17.83% (Supplementary Materials, Table S1). The results show that the method has good repeatability, stability and reliability for metabolomics analysis.

### 3.4. Qualitative Analysis of Liver Metabolites

Liver samples were analyzed by UPLC-Orbitrap-MS/MS, and the details of this process are shown in Figure 3(a). Complicated raw data were obtained and displayed as the total ion chromatogram (TIC) shown in Fig. S2. Effective mass spectra of peaks were extracted by CD software and matched with accurate mass data, isotope patterns, and mass databases. For example, taurocholic acid [M + H] + at m/z 516.2969 and m/z 517.3000 and m/z 518.2989 of its isotope were found in MS1 (Fig. S3a). Then, the MS/MS of taurocholic acid was matched to the mzCloud library, and a score was given to indicate the similarity (Fig. S3b). In this research, we chose the mzCloud score of peaks greater than 75 and could match ChemSpider, mzVault, or Masslist. Finally, a total of 133 peaks were identified in each sample, and the Human Metabolome Database (HMDB) and Kyoto Encyclopedia of Genes and Genomes (KEGG) were used for further confirmation of metabolites. Detailed information on the metabolites is presented in Table S1. In addition, the chromatographic peak area of those metabolites was normalized before multivariate analysis.

### 3.5. Multivariate Statistical Analysis and Potential Metabolites

**Figure 4:**
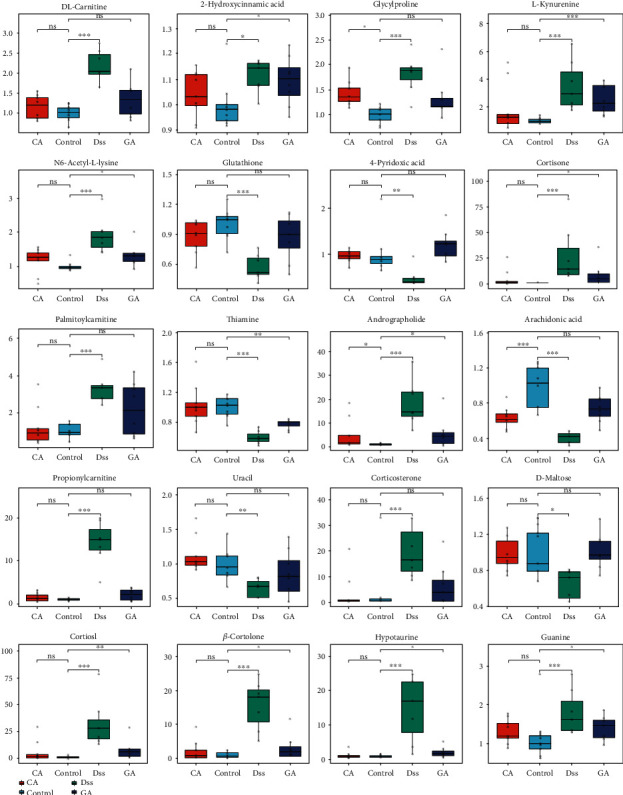
Box-plot of potential metabolites in the four groups: control, DSS, CA and GA group (∗*P* < 0.05, ∗∗*P* < 0.01, ∗∗∗*P* < 0.001, ns means not significant).

Based on the qualitative and semiquantitative results of liver metabolites, chemometric methods were utilized to provide more in-depth information about them. The PCA results (Figure 3(b)) revealed that metabolites in the different groups were separated into distinct clusters, and dots of two treatment groups were scattered in the middle between the DSS and control groups. The differential metabolites among diverse groups with *p* < 0.05 were selected by one-way analysis of variance (one-way ANOVA), as shown in Fig. S4a and Table S2. Heatmap analysis was performed to represent the differences and similarities among the four groups, as shown in Figure 3(c). The results suggested that some metabolite levels may be reversed by the cecropin A or gentamicin treatment. Then, the OPLS-DA model and S-plot were established to screen potential metabolites using restrictions including VIP>1 and the absolute value of the correlation coefficient (Corr) >0.80. Finally, 20 compounds were selected by this limiting condition and were tagged using red dots in the S-plot shown in Fig. S4b–d. These potential metabolites were considered biomarkers, and the details of those metabolites are listed in Table 1. The relative concentration changes in the 4 groups are shown in Figure 4. Overall, components related to glutathione metabolism, energy metabolism and inflammation, such as glutathione, maltose and arachidonic acid, as well as thiamine and 4-pyridoxic acid, which are metabolites of the liver, were downregulated in the DSS group and upregulated in the CA and GA group. Conversely, steroid hormones metabolites such as cortisol and cortisone were upregulated because of the long-term stress resulting from IBD and were alleviated by treatment with CA and GA.

### 3.6. Metabolic Pathway and Potential Therapeutic Targets

**Figure 5:**
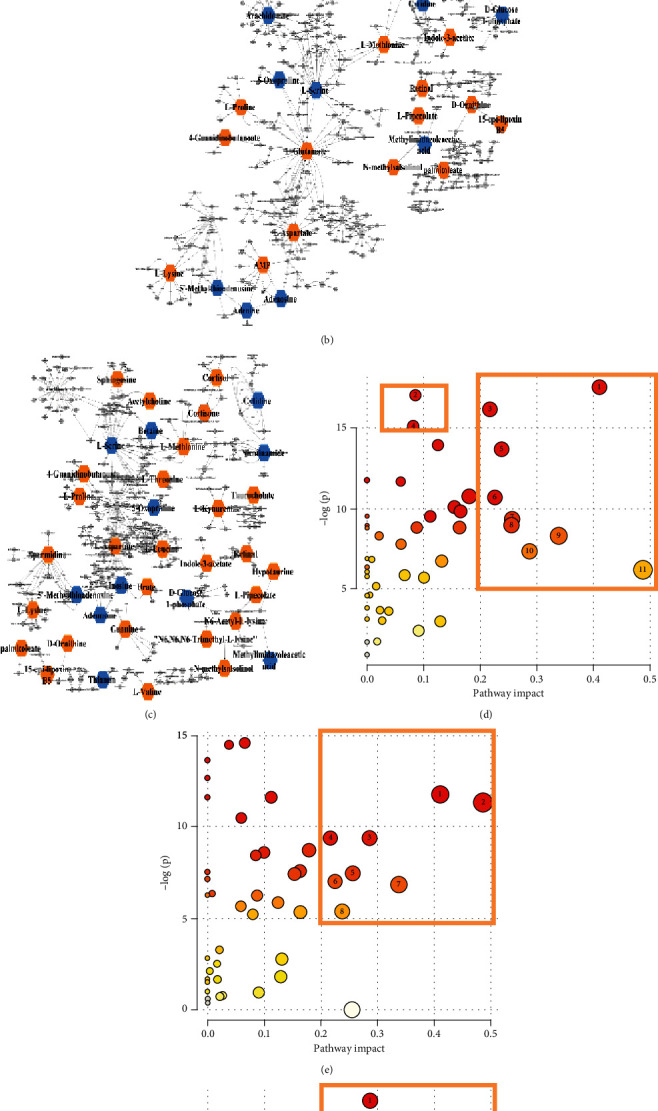
Metabolism network analysis of three treatment groups: (a) DSS group vs control group. (b) CA group vs control group. (c) GA group vs control group. Blue indicates downregulated metabolites, and red indicates upregulated metabolites. Metabolism pathway analysis of the four groups. The *x*-axis and the *y*-axis represent the pathway impact and enrichment, respectively. The critical pathways are framed by yellow boxes. (d) DSS vs control, 1-taurine and hypotaurine metabolism; 2-steroid hormone biosynthesis; 3-arachidonic acid metabolism; 4-tryptophan metabolism; 5-cysteine and methionine metabolism; 6-aminoacyl-tRNA biosynthesis; 7-glycine, serine and threonine metabolism; 8-retinol metabolism; 9-glutathione metabolism; 10-arginine and proline metabolism; 11-alanine, aspartate and glutamate metabolism. (e) DSS vs CA, 1-taurine and hypotaurine metabolism; 2-alanine, aspartate and glutamate metabolism; 3-glutathione metabolism; 4-arachidonic acid metabolism; 5-glycine, serine and threonine metabolism; 6-aminoacyl-tRNA biosynthesis; 7-arginine and proline metabolism; 8-cysteine and methionine metabolism. (f) DSS vs GA, 1-glutathione metabolism; 2-arachidonic acid metabolism; 3-arginine and proline metabolism; 4-alanine, aspartate and glutamate metabolism; 5-glycine, serine and threonine metabolism; 6-aminoacyl-tRNA biosynthesis; 7-taurine and hypotaurine metabolism; 8-cysteine and methionine metabolism.

**Figure 6:**
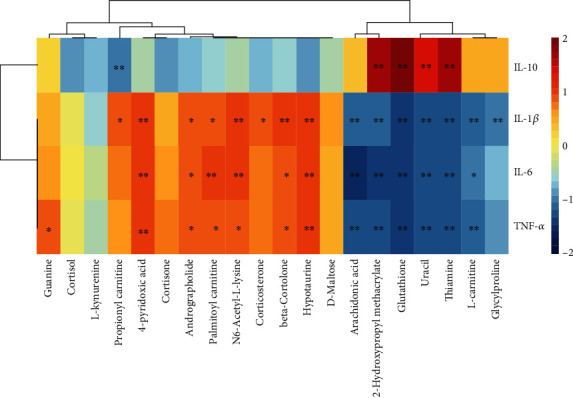
The Spearman correlation analysis among cytokines and biomarkers, and clustering is shown as a heat map. Color from red to blue indicates a positive correlation to a negative correlation, and the asterisk represents the significance of the correlation, ∗*P* < 0.05, ∗∗*P* < 0.01.

In this research, a metabolic network analysis was established based on the p-value and fold-change among the different groups. There were 21 downregulated compounds and 22 upregulated compounds in the metabolic network of the DSS group (Figure 5(a)). In the GA group (Figure 5(b)), 11 compounds were downregulated and 27 compounds were upregulated, whereas only 9 compounds were downregulated and 16 were upregulated in the CA group (Figure 5(c)). Additionally, corresponding to the metabolic network, the metabolic pathway was analyzed on the Metaboanalyst 4.0 platform. Figures 5(d)–5(f) shows the pathway analysis between the DSS and control group, DSS and CA group, and the DSS and GA group. There were extensive changes in the pathways of liver metabolism influenced by IBD. On the basis of an impact >0.2 and -log(p) >5, Figure 5(d) shows that the pathways with significant impacts were taurine and hypotaurine metabolism; arachidonic acid metabolism; cysteine and methionine metabolism; aminoacyl-tRNA biosynthesis; glycine, serine and threonine metabolism; arginine and proline metabolism; glutathione metabolism; and alanine, aspartate and glutamate metabolism. Additionally, steroid hormone biosynthesis and tryptophan metabolism were impacted. Figures 5(e) and 5(f) shows that the predominant pathway of liver metabolism with the CA and GA treatment is through its effects on amino acid metabolism, aminoacyl-tRNA biosynthesis, and especially a regulating effect on taurine and hypotaurine metabolism, glutathione metabolism, and arachidonic acid metabolism. In addition, a Spearman correlation analysis showed the correlation between cytokines and biomarkers, as shown in Figure 6. Most of the 20 biomarkers were positively or negatively correlated with four cytokines, among which thiamine, uracil, glutathione, and 2-hydroxypropyl methacrylate were significantly correlated with proinflammatory (TNF-*α*, IL-1*β*, and IL-6) and anti-inflammatory (IL-10) cytokines, and those metabolites also belong to the pathway regulated by CA and GA, implying that those biomarkers may act as potential therapeutic targets of liver disorder resulting from IBD.

## 4. Discussion

IBD has a complex pathology and easily causes systemic inflammatory responses. In our results, we established a refined and translationally relevant model of DSS chronic colitis in C57BL/6 mice and found morbidity in liver morphology and significant increases in proinflammatory cytokines (TNF-*α*, IL-1*β*, and IL-6) in the liver tissue of IBD mice. Metabolomics revealed 20 potential metabolites closely related to inflammatory cytokines, mainly involved in the following aspects: (a) bile acid metabolism disorder (taurine, taurocholate, hypotaurine); (b) arachidonic acid metabolism disorder; (c) amino acid and protein metabolism (guanine, glutathione, glutamate acid, glycylproline, kynurenine); and (d) steroid hormone biosynthesis (cortisol, cortisone, corticosterone).

Bile acids are liver-derived cholesterol derivatives that control digestion and modulate lipid metabolism [17]. Many published studies have suggested the importance of appropriately maintaining bile acid homeostasis to liver metabolism, and bile acid overload will cause inflammation and impair the liver [18–20]. Furthermore, recent research has shown that the gut microbiota plays a key role in various disorders within and beyond the gastrointestinal tract [21, 22]. The gut microbiota affects intestinal signaling and enterohepatic circulation of bile acids via a “liver-microbiome axis” [7, 23]. In this research, bile acid metabolism disorders may result from disturbances of the gut microbiota due to IBD.

Arachidonic acid is a polyunsaturated fatty acid that is essential for normal health and is a component of the biological cell membrane, which can maintain the normal permeability and flexibility of cells [24]. Notably, during inflammation, arachidonic acid reacts with enzymes to form prostaglandins, leukotrienes, and thromboxanes, which are considered predominantly proinflammatory molecules [25]. Additionally, the arachidonic acid metabolic pathway was changed because phospholipase has been shown to be decreased in patients with IBD [26]. Therefore, arachidonic acid was downregulated in the liver during the course of IBD.

The liver is the major metabolic organ for amino acids and proteins. The DSS group was altered in amino acid and protein metabolism, and amino acid-related compounds such as pyroglutamic acid, glutamic acid, and glutathione were involved in glutathione metabolism and downregulated in the DSS group. The glutathione pathway is a key hepatic defense mechanism and deactivates reactive metabolites before they have an opportunity to damage cellular proteins [27]. In addition, we found that tryptophan metabolism was influenced considerably by IBD in our research. Tryptophan is an essential amino acid involved in various biological processes, and its metabolites play a crucial role in the regulation of immunity [28]. Recently published studies have shown that gut microbiota can affect host physiology and pathology by interfering with tryptophan metabolism [29]. Therefore, in the process of IBD, the disorder of tryptophan metabolism in the liver may be caused by the intervention mechanism of gut microbiota on tryptophan metabolism.

IBD also caused the disarray of steroid hormone biosynthesis. Glucocorticoids are the primary steroid hormones, including cortisol and cortisone, which are related factors that indicate stress and pain and play a decisive role in metabolism, maintaining energy balance and animal survival in adversity [30]. An appropriate stress response is conducive to the body's short-term adaptation to the environment; however, long-term stress or strong stimulation and a high level of glucocorticoids are bound to cause energy metabolism and hormone secretion disorder, thereby affecting health [31]. Published research has found that excessive cortisol secretion will promote the catabolism of proteins in extrahepatic tissues, resulting in negative nitrogen balance [32]. In our study, IBD exposed mice to long-term stress and promoted excessive glucocorticoid secretion (cortisol and cortisone, as shown in Figure 4), which may induce liver injury.

The gut microflora is an important factor in regulating gastrointestinal homeostasis, affecting the immune system and host metabolism [33]. In recent studies, the gut microflora has been closely related to the development of hepatopathy [34]. In our studies, we found that cecropin A has better effectiveness than gentamicin, and CA reversed bile acid metabolism and amino acid and protein and steroid hormone biosynthesis disarray caused by IBD. The concentrations of taurine, taurocholate, hypotaurine, cortisol, cortisone, and corticosterone were downregulated in the CA group. Glutathione and metabolites of tryptophan metabolism returned to normal levels. Compared to CA, the reverse ability of the GA group was weak, and only glutathione and arachidonic acid levels were reversed. The possible reasons for these results were that cecropin A can improve the abundance of beneficial bacteria and reduce the adhesion of harmful bacteria to cells [35], which may benefit intestinal epithelium recovery and regulate gut microbiota, thereby alleviating IBD and liver metabolism disturbance. On the other hand, cecropin A and gentamicin showed different effects on their microbiota populations, which may lead to different degrees of recovery from intestinal injury.

## 5. Conclusion

This study concentrates on revealing the metabolic profiles of the liver in mice with DSS-induced IBD. Metabolomics analysis was performed on UPLC-HRMS combined with effective untargeted qualitative tools, and 20 potential metabolites were screened as biomarkers to represent the characteristics of liver disorder in IBD. Metabolic pathway analysis and metabolite network analysis indicated that bile acid metabolism, arachidonic acid metabolism, amino acid and protein metabolism, and steroid hormone biosynthesis were changed in the liver caused by IBD. Additionally, the correlation analysis between cytokines (TNF-*α*, IL-1*β*, IL-6, and IL-10) and biomarkers indicated that CA and GA may be able to regulate those metabolites and metabolic pathways by regulating the structure of gut microflora in the treatment of IBD, thereby assisting in alleviating liver inflammation. We hope that our efforts may serve as a reference in the study of IBD and its associated molecular mechanisms in the liver.

## Figures and Tables

**Table 1:** The twenty significant metabolites in liver of control group, DSS group, CA group, and GA group.

No.	Name	t_R_(min)	HMDB	KEGG	VIP>1	Relative intensity			
						Control	DSS	CA	GA
1	Propionyl carnitine	2.31	HMDB0000824	C03017	∆^a^	384.21 ± 98.64	5700.72 ± 2028.84^c^	588.66 ± 375.78	847.76 ± 530.07
2	Arachidonic acid	14.09	HMDB0001043	C00219	∆	5997.26 ± 1389.78	2527.74 ± 396.91^c^	3919.27 ± 744.50^e^	4579.15 ± 951.61
3	Hypotaurine	1.23	HMDB0000965	C00519	∆	284.55 ± 70.53	4235.41 ± 2659.97^c^	358.70 ± 289.65	606.52 ± 428.58^f^
4	D-maltose	1.21	HMDB0000163	C00208	∆	1779.42 ± 483.21	1155.87 ± 283.50^b^	1747.31 ± 317.29	1807.18 ± 343.14
5	4-Pyridoxic acid	2.02	HMDB0000017	C00847	∆	144.45 ± 66.08	65.88 ± 29.11^b^	131.24 ± 19.31	164.84 ± 46.90
6	Cortisol	8.62	HMDB0000063	C00735	∆	11.69 ± 9.99	506.20 ± 352.53^c^	21.59 ± 20.54	73.07 ± 52.63^g^
7	Guanine	1.34	HMDB0000132	C00242	∆	187.17 ± 47.36	336.74 ± 109.82^c^	245.23 ± 58.58	263.57 ± 57.07^f^
8	Uracil	1.35	HMDB0000300	C00106	∆	4359.67 ± 764.58	2963.04 ± 608.96^b^	5243.61 ± 1162.11	3965.74 ± 1520.60
9	L-Carnitine	1.24	HMDB0000062	C00318	∆	17807.48 ± 3512.00	38793.25 ± 6811.27^c^	20469.79 ± 5089.24	23672.54 ± 7816.16
10	*β*-Cortolone	8.32	HMDB0013221	C05481	∆	18.50 ± 12.94	234.38 ± 106.55^c^	5.97 ± 4.67	28.75 ± 24.15^f^
11	Corticosterone	8.08	HMDB0001547	C02140	∆	3.72 ± 2.15	73.06 ± 37.02^c^	2.27 ± 0.84	37.94 ± 30.86
12	Andrographolide	9.62	Na∗	NA	∆	2.31 ± 0.62	42.36 ± 22.28^c^	4.05 ± 3.04	8.07 ± 5.67
13	Glycylproline	1.70	HMDB0000721	NA	∆	116.59 ± 19.36	213.04 ± 45.74^c^	164.62 ± 29.84^d^	154.76 ± 49.39
14	Thiamine	1.18	HMDB0000235	C00378	∆	1213.24 ± 172.57	721.46 ± 104.90^c^	1244.25 ± 330.44	936.37 ± 77.03^g^
15	Glutathione	1.33	HMDB0000125	C00051	—	39617.25 ± 6036.10	22513.56 ± 4980.63^c^	34831.89 ± 6374.22	34240.51 ± 8982.61
16	N6-acetyl-L-lysine	1.28	HMDB0000206	C02727	∆	265.83 ± 37.06	507.29 ± 141.26^c^	309.20 ± 95.39	350.36 ± 83.69^f^
17	L-Kynurenine	3.25	HMDB0000684	C00328	∆	654.22 ± 129.20	2302.36 ± 1173.26^c^	1233.12 ± 1104.86	1653.55 ± 699.91^g^
18	2-Hydroxypropyl methacrylate	6.68	NA	NA	—	13422.12 ± 3602.44	10060.25 ± 2172.58^c^	11174.65 ± 1769.03	8035.20 ± 2318.02^g^
19	Palmitoyl carnitine	11.76	HMDB0000222	C02990	∆	39.64 ± 15.63	131.29 ± 32.20^c^	39.09 ± 24.66	87.65 ± 56.63
20	Cortisone	8.38	HMDB0002802	C00762	∆	1.81 ± 0.46	33.18 ± 27.09^b^	2.02 ± 0.92	10.02 ± 8.24^f^

∗NA means not available. ^a^∆ means VIP>1 in OPLS-DA. ^b^DSS group compared with control group, *p* < 0.05.^c^DSS group compared with control group, *p* < 0.01.^d^CA group compared with control group, *p* < 0.05.^e^CA group compared with control group, *p* < 0.01. ^f^GA group compared with control group, *p* < 0.05. ^g^GA group compared with control group, *p* < 0.01.

## Data Availability

The data used to support the findings of this study are available from the corresponding author upon request.

## Additional Points

*Preprint*. This work has a preprint online, preprint link: https://www.researchsquare.com/article/rs-7302/v1, DOI: 10.21203/rs.2.16664/v1.

## Supplementary Materials

Supplementary Materials Supplementary Figure 1 Histopathological results of colon and liver stained with H&E of the control group (a and b) and DSS group (c and d). Supplementary Figure 2: The total ion chromatograms (TICs) of the control group, DSS group, CA group and GA group. Supplementary Figure 3: Two steps used for metabolite identification: (a) isotope pattern matching and (b) MS/MS spectral library matching. Supplementary Figure 4: Selection of potential metabolites based on one-way ANOVA and OPLS-DA. (a) One-way ANOVA of metabolites in the four groups; red dots represent metabolites with *p* < 0.05, and the green dot represents *p* > 0.05. (b) OPLS-DA between the DSS group and the control group. (c) Permutation test to evaluate OPLS-DA. (d) S-plot showing the OPLS-DA analysis of the DSS group and control group. Potential biomarkers (red dots) were chosen according to *p* < 0.05 in the one-way ANOVA, VIP>1 in OPLS-DA, and the absolute value of the correlation coefficient (Corr) >0.80 of the S-plot. Supplementary Table 1: List of metabolites identified in the liver. Supplementary Table 2: List of the 52 metabolites that were screened by one-way analysis of variance of metabolites in the control group, DSS group, CA group and GA group.
