# Supplementary material for: Metabolic Profiling by UPLC–Orbitrap–MS/MS of Liver from C57BL/6 Mice with DSS-Induced Inflammatory Bowel Disease
Source: Mediators Inflamm. 2020 Sep 23;2020:6020247. doi: 10.1155/2020/6020247 (PMC7530511; doi:10.1155/2020/6020247)
Supplement: Supplementary Materials — Supplementary Figure 1 Histopathological results of colon and liver stained with H&E of the control group (a and b) and DSS group (c and d). Supplementary Figure 2: The total ion chromatograms (TICs) of the control group, DSS group, CA group and GA group. Supplementary Figure 3: Two steps used for metabolite identification: (a) isotope pattern matching and (b) MS/MS spectral library matching. Supplementary Figure 4: Selection of potential metabolites based on one-way ANOVA and OPLS-DA. (a) One-way ANOVA of metabolites in the four groups; red dots represent metabolites with p < 0.05, and the green dot represents p > 0.05. (b) OPLS-DA between the DSS group and the control group. (c) Permutation test to evaluate OPLS-DA. (d) S-plot showing the OPLS-DA analysis of the DSS group and control group. Potential biomarkers (red dots) were chosen according to p < 0.05 in the one-way ANOVA, VIP>1 in OPLS-DA, and the absolute value of the correlation coefficient (Corr) >0.80 of the S-plot. Supplementary Table 1: List of metabolites identified in the liver. Supplementary Table 2: List of the 52 metabolites that were screened by one-way analysis of variance of metabolites in the control group, DSS group, CA group and GA group. [file 6020247.f1.docx]

**Supplementary Information**

**Metabolic profiling by UPLC–Orbitrap–MS/MS of liver from C57BL/6 mice with DSS-induced inﬂammatory bowel disease** Zhongquan Xin,^1^ Zhenya Zhai,^2^ Hongrong Long,^1^ Fan Zhang,^1^ Xiaojun Ni,^3^ Jinping Deng,^1^ Lunzhao Yi,^4^ and Baichuan Deng^5^

^1^Guangdong Provincial Key Laboratory of Animal Nutrition Control, National Engineering Research Center for Breeding Swine Industry, College of Animal Science, Subtropical Institute of Animal Nutrition and Feed, South China Agricultural University, Guangzhou, China

^2^Institute of Biological Resource, Jiangxi Academy of Sciences, Nanchang, 330029, China

^3^Yunnan Animal Science and Veterinary Institute, Jindian, Panlong County, Kunming City, Yunnan Province, China

^4^Yunnan Food Safety Research Institute, Kunming University of Science and Technology, Kunming, 650500, China

^5^Maoming Branch, Guangdong Laboratory for Lingnan Modern Agriculture, Guangdong Provincial Key Laboratory of Animal Nutrition Control, National Engineering Research Center for Breeding Swine Industry, College of Animal Science, South China Agricultural University, Guangzhou, Guangdong, P.R. China

Correspondence should be addressed to Lunzhao Yi; [yilunzhao@kmust.edu.cn](mailto:yilunzhao@kmust.edu.cn) and Baichuan Deng; dengbaichuan@scau.edu.cn

**Figure** **legends**

**Fig. S1**

Histopathological results of colon and liver stained with H&E of the control group (a and b) and DSS group (c and d).

**Fig. S2**

The total ion chromatograms (TICs) of the control group, DSS group, CA group and GA group.

**Fig. S3**

Two steps used for metabolite identification: (a) isotope pattern matching and (b) MS/MS spectral library matching.

**Fig. S4**

Selection of potential metabolites based on one-way ANOVA and OPLS-DA. (a) One-way ANOVA of metabolites in the four groups; red dots represent metabolites with p<0.05, and the green dot represents p >0.05. (b) OPLS-DA between the DSS group and the control group. (c) Permutation test to evaluate OPLS-DA. (d) S-plot showing the OPLS-DA analysis of the DSS group and control group. Potential biomarkers (red dots) were chosen according to p < 0.05 in the one-way ANOVA, VIP > 1 in OPLS-DA, and the absolute value of the correlation coefficient (Corr) >0.80 of the S-plot.

**Table legends**

**Table S1**

Lists of metabolites identified in liver

**Table S2**

List of the 52 metabolites which were screened by one-way analysis of variance of metabolites in control group, DSS group, CA group and GA group


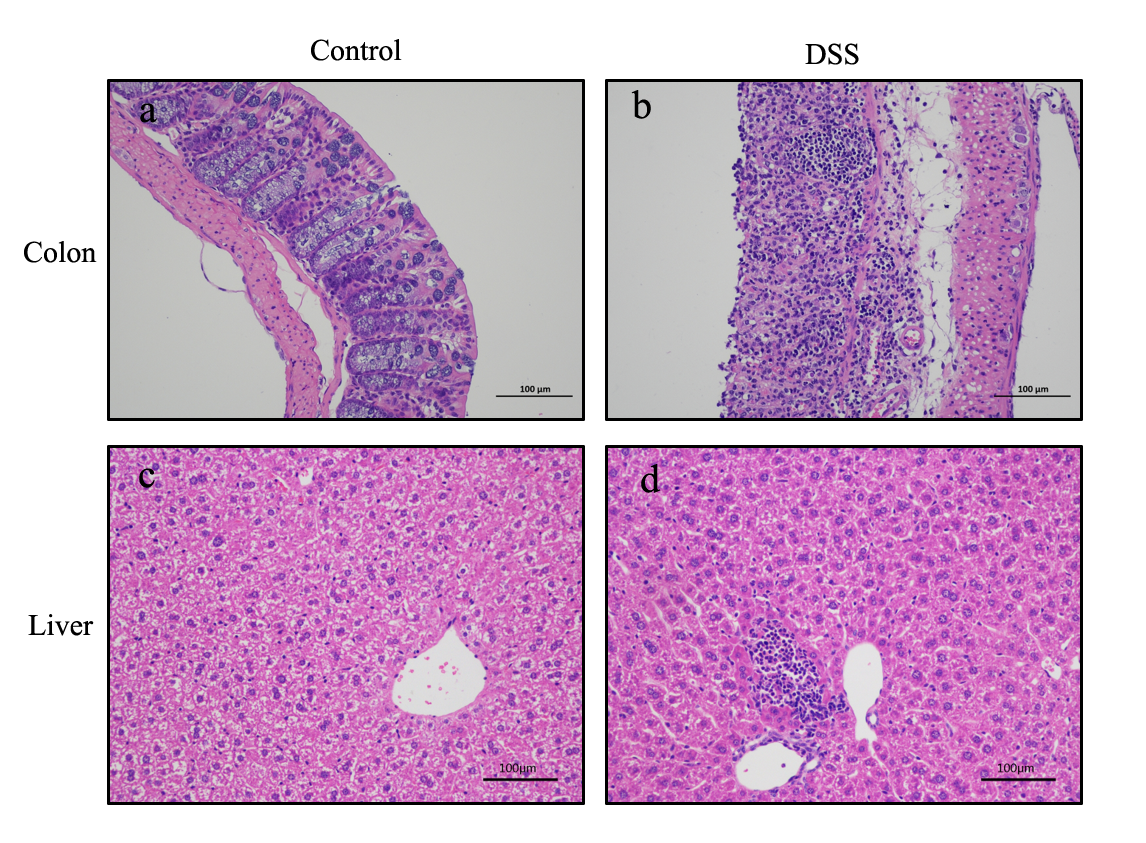


Fig.S1


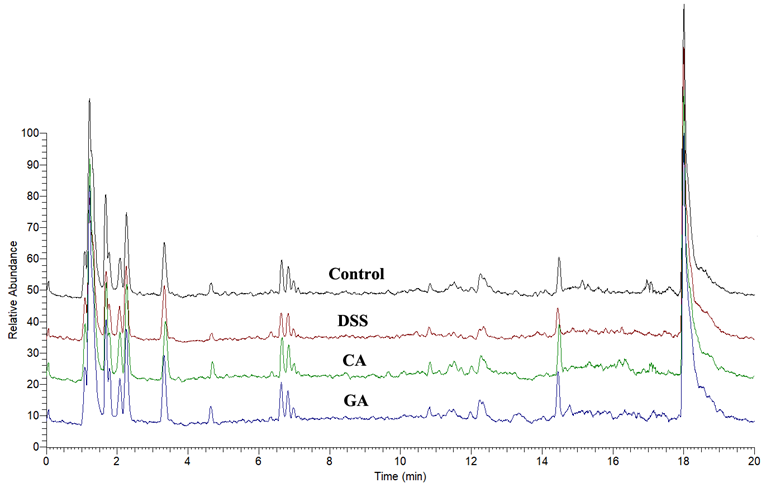


Fig. S2


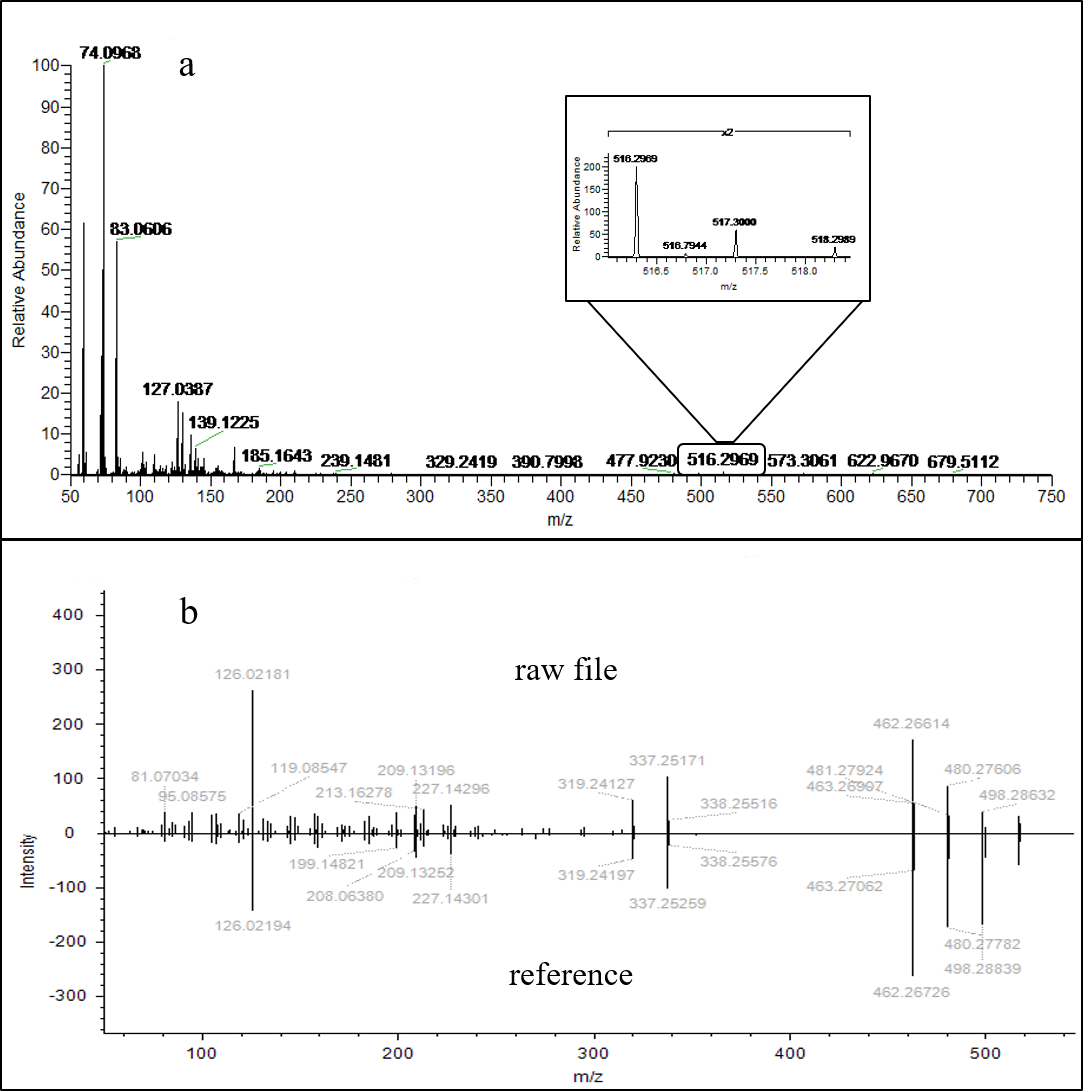


Fig. S3


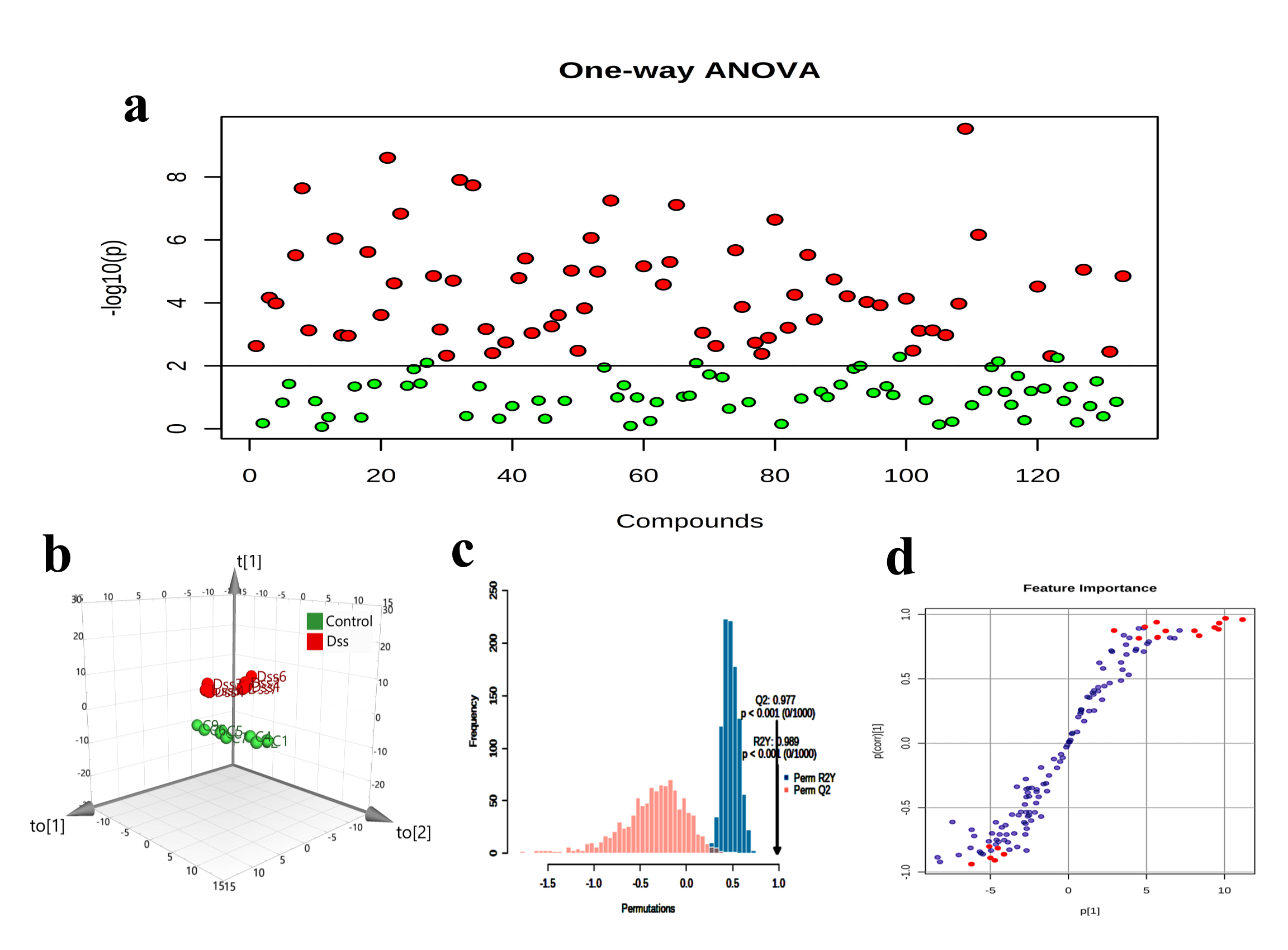


Fig. S4

Table S1. Lists of metabolites identified in liver.

| NO. | Name | t_R_[min] | MW | Formula | VIP | HMDB | KEGG | QC-mean±SD | QC-CV (%) |
| --- | --- | --- | --- | --- | --- | --- | --- | --- | --- |
|  | 11-Dehydrocorticosterone | 8.96 | 344.1975 | C_22_H_43_NO | 1.7226 | HMDB0004029 | C05490 | 2.59±0.19 | 7.14 |
|  | 12-Aminododecanoicacid | 8.63 | 215.1878 | C_12_H_25_NO_2_ | 0.2322 | NA^*^ | NA | 1.81±0.14 | 7.68 |
|  | 1-Linoleoylglycerol | 11.37 | 354.2754 | C_21_H_34_O_5_ | 0.4088 | HMDB0011568 | NA | 4.77±0.80 | 16.83 |
|  | 1-Methylhistidine | 1.21 | 169.0845 | C_7_H_11_N_3_O_2_ | 1.6566 | HMDB0000001 | C01152 | 4.38±0.75 | 17.21 |
|  | 1-Tetradecylamine | 10.53 | 213.2449 | C_14_H_31_N | 1.6027 | NA | NA | 6.96±0.51 | 7.38 |
|  | 2-Aminooctadec-4-yne-13-diol | 10.65 | 297.2657 | C_18_H_35_NO_2_ | 0.4480 | NA | NA | 24.74±2.24 | 9.04 |
|  | 2-Ethylamino-1-phenylbutane | 10.86 | 177.1511 | C_12_H_19_N | 0.5981 | NA | NA | 162.50±11.50 | 7.08 |
|  | 2-Hydroxycinnamicacid | 1.80 | 164.0467 | C_9_H_10_O_4_ | 1.0797 | HMDB0002641 | C01772 | 4241.86±411.88 | 9.71 |
|  | 2-Hydroxypropylmethacrylate | 6.68 | 144.0783 | C_7_H_12_O_3_ | 0.7010 | NA | NA | 1268.38±72.90 | 5.75 |
|  | 34-Dihydroxyphenylpropionicacid | 1.40 | 182.0579 | C_9_H_10_O_4_ | 0.6383 | HMDB0000423 | C10447 | 1594.57±141.57 | 8.88 |
|  | 35-di-tert-Butyl-4-hydroxybenzaldehyde | 11.83 | 234.1610 | C_15_H_22_O_2_ | 0.4697 | NA | NA | 36.78±2.84 | 7.73 |
|  | 3'-Adenosinemonophosphate(3'-AMP) | 1.33 | 347.0618 | C_21_H_30_O_4_ | 0.0356 | HMDB0003540 | C01367 | 57.97±4.88 | 8.43 |
|  | 3-Hydroxypicolinicacid | 1.99 | 139.0265 | C_6_H_5_NO_3_ | 0.3070 | HMDB0013188 | C18620 | 28.12±3.24 | 11.51 |
|  | 3-Methylindole | 4.71 | 131.0731 | C_9_H_9_N | 1.9113 | HMDB0000466 | C08313 | 14.96±1.49 | 9.96 |
|  | 4-Guanidinobutyricacid | 1.33 | 145.0846 | C_5_H_11_N_3_O_2_ | 0.2245 | HMDB0003464 | C01035 | 110.30±6.12 | 5.55 |
|  | 4-Indolecarbaldehyde | 4.71 | 145.0523 | C_9_H_7_NO | 1.1363 | HMDB0029737 | C08493 | 36.26±3.59 | 9.89 |
|  | 4-Phenyl-3-buten-2-one | 8.53 | 146.0726 | C_10_H_10_O | 0.1978 | HMDB0031617 | NA | 16.81±1.28 | 7.60 |
|  | 4-Pyridoxicacid | 2.02 | 183.0526 | C_8_H_9_NO_4_ | 1.1417 | HMDB0000017 | C00847 | 12.73±1.81 | 14.20 |
|  | 5-Hydroxyindole | 6.48 | 133.0523 | C_8_H_7_NO | 1.2247 | HMDB0059805 | NA | 1.04±0.11 | 10.24 |
|  | 5-Hydroxyindole-3-aceticacid | 3.26 | 191.0576 | C_10_H_9_NO_3_ | 1.3794 | HMDB0000763 | C05635 | 181.54±31.82 | 17.53 |
|  | 5'-Methylthioadenosine | 4.30 | 297.0885 | C_11_H_15_N_5_O_3_S | 2.0259 | HMDB0001173 | C00170 | 46.41±5.53 | 11.93 |
|  | 6-Methylquinoline | 5.22 | 143.0730 | C_10_H_9_N | 0.7632 | HMDB0033115 | NA | 0.43±0.06 | 14.89 |
|  | 7-Methylguanosine | 2.10 | 297.1063 | C_11_H_15_N_5_O_5_ | 1.0380 | HMDB0001107 | NA | 9.14±0.51 | 5.58 |
|  | 9-OxoODE | 12.07 | 294.2183 | C_18_H_30_O_3_ | 0.4602 | HMDB0004669 | C14766 | 5.38±0.49 | 9.07 |
|  | Acetylcholine | 1.27 | 145.1098 | C_7_H_15_NO_2_ | 0.9837 | HMDB0000895 | C01996 | 3.92±0.59 | 15.16 |
|  | Acetyl-*β*-methylcholine | 1.33 | 159.1254 | C_8_H_17_NO_2_ | 0.4188 | HMDB0015654 | C07471 | 107.01±6.34 | 5.92 |
|  | Adenine | 1.30 | 135.0540 | C_5_H_5_N_5_ | 0.6739 | HMDB0000034 | C00147 | 337.33±22.18 | 6.58 |
|  | Adenosine | 1.70 | 267.0958 | C_10_H_13_N_5_O_4_ | 1.3600 | HMDB0000050 | C00212 | 33.34±5.33 | 16.00 |
|  | Adenosine5'-monophosphate | 4.52 | 347.0619 | C_10_H_14_N_5_O_7_P | 0.5601 | HMDB0000045 | C00020 | 33.14±2.30 | 6.94 |
|  | Adrenicacid | 15.06 | 332.2703 | C_22_H_36_O_2_ | 0.9569 | HMDB0002226 | C16527 | 28.35±1.01 | 3.58 |
|  | Andrographolide | 9.62 | 332.1976 | C_20_H_30_O_5_ | 1.9006 | NA | NA | 4.56±0.68 | 14.91 |
|  | Arachidonic acid | 14.09 | 304.2389 | C_20_H_32_O_2_ | 1.4701 | HMDB0001043 | C00219 | 1.61±0.21 | 13.18 |
|  | Asparagine | 1.21 | 132.0530 | C_4_H_8_N_2_O_3_ | 0.4017 | HMDB0000168 | C00152 | 427.89±33.41 | 7.81 |
|  | Asymmetricdimethylarginine | 1.22 | 202.1423 | C_8_H_18_N_4_O_2_ | 1.0467 | HMDB0001539 | C03626 | 275.63±20.16 | 7.31 |
|  | Benzophenone | 10.90 | 182.0727 | C_13_H_10_O | 0.4691 | HMDB0032049 | C06354 | 204.44±11.65 | 5.70 |
|  | Benzotriazole | 5.80 | 119.0482 | C_6_H_5_N_3_ | 0.0085 | NA | NA | 14.73±1.16 | 7.88 |
|  | Betaine | 1.25 | 117.0787 | C_5_H_11_NO_2_ | 1.4749 | HMDB0000043 | C00719 | 15.79±2.20 | 13.96 |
|  | Caprolactam | 4.87 | 113.0840 | C_6_H_11_NO | 0.3576 | HMDB0062769 | C06593 | 4401.22±159.60 | 3.63 |
|  | Choline | 1.22 | 103.0997 | C_5_H_13_NO | 0.8600 | HMDB0000097 | C00114 | 31.89±5.06 | 15.86 |
|  | Cis-stilbeneoxide | 11.32 | 196.0883 | C_14_H_12_O | 0.6494 | HMDB0059631 | C16014 | 1586.61±63.52 | 4.00 |
|  | Corticosterone | 8.08 | 346.2132 | C_21_H_28_O_4_ | 2.2513 | HMDB0001547 | C02140 | 2.64±0.30 | 11.37 |
|  | Cortisol | 8.62 | 362.2080 | C_21_H_30_O_5_ | 2.6231 | HMDB0000063 | C00735 | 2.52±0.20 | 7.74 |
|  | Cortisone | 8.38 | 360.1924 | C_21_H_28_O_5_ | 1.9028 | HMDB0002802 | C00762 | 16.70±1.18 | 7.04 |
|  | Creatine | 1.26 | 131.0691 | C_4_H_9_N_3_O_2_ | 0.5857 | HMDB0000064 | C00300 | 1.68±0.13 | 7.49 |
|  | Cyclohexylamine | 2.98 | 82.0784 | C_6_H_13_N | 0.5622 | HMDB0031404 | C00571 | 1000.54±34.14 | 3.41 |
|  | Cytidine | 1.28 | 243.0846 | C_9_H_13_N_3_O_5_ | 0.8924 | HMDB0000089 | C00475 | 161.89±26.94 | 16.64 |
|  | Cytosine | 1.28 | 111.0432 | C_4_H_5_N_3_O | 0.7514 | HMDB0000630 | C00380 | 62.68±5.05 | 8.05 |
|  | Daidzein | 7.78 | 254.0569 | C_15_H_10_O_4_ | 0.4528 | HMDB0003312 | C10208 | 103.72±7.03 | 6.78 |
|  | L-Carnitine | 1.24 | 161.1046 | C_7_H_15_NO_3_ | 1.3212 | HMDB0000062 | C00318 | 2.72±0.29 | 10.73 |
|  | L-Stachydrine | 1.30 | 143.0941 | C_7_H_13_NO_2_ | 1.5429 | NA | NA | 2043.05±71.07 | 3.48 |
|  | L-Tryptophan | 4.71 | 204.0891 | C_11_H_12_N_2_O_2_ | 0.2046 | HMDB0013609 | C00525 | 54.36±8.48 | 15.60 |
|  | D-Maltose | 1.21 | 364.0968 | C_12_H_22_O_11_ | 1.1307 | HMDB0000163 | C00208 | 1949.46±282.33 | 14.48 |
|  | Docosapentaenoic acid | 14.22 | 330.2547 | C_22_H_34_O_2_ | 1.2645 | HMDB0006528 | C16513 | 137.42±11.53 | 8.39 |
|  | D-Raffinose | 1.21 | 526.1491 | C_18_H_32_O_16_ | 0.8539 | HMDB0003213 | C00492 | 25.23±1.20 | 4.74 |
|  | Eicosapentaenoic acid | 12.05 | 302.2234 | C_20_H_32_O_3_ | 1.2404 | HMDB0001999 | C06428 | 104.36±7.02 | 6.73 |
|  | Erucamide | 16.64 | 337.3329 | C_21_H_38_O_4_ | 1.0240 | NA | NA | 148.36±18.81 | 12.68 |
|  | Ethyllevulinate | 4.55 | 144.0782 | C_7_H_12_O_3_ | 0.6167 | NA | NA | 4.58±0.64 | 13.86 |
|  | Flurandrenolide | 5.61 | 436.2269 | C_24_H_33_FO_6_ | 0.0177 | HMDB0014984 | NA | 8.72±0.65 | 7.45 |
|  | Folinicacid | 4.14 | 473.1644 | C_20_H_23_N_7_O_7_ | 0.9469 | HMDB0001562 | C03479 | 22.63±2.95 | 13.04 |
|  | Glucose1-phosphate | 1.26 | 260.0287 | C_6_H_13_O_9_P | 1.2803 | HMDB0001586 | C00103 | 23.96±2.19 | 9.15 |
|  | Glutamylglutamic acid | 1.31 | 276.0948 | C_10_H_16_N_2_O_7_ | 0.0210 | HMDB0028818 | C01425 | 34.64±6.17 | 17.81 |
|  | Glycylleucine | 4.28 | 188.1155 | C_8_H_16_N_2_O_3_ | 0.4773 | HMDB0000759 | C02155 | 48.39±4.41 | 9.11 |
|  | Glycylproline | 1.70 | 172.0843 | C_7_H_12_N_2_O_3_ | 1.1102 | HMDB0000721 | NA | 8.30±1.25 | 15.12 |
|  | Guanine | 1.34 | 151.0488 | C_5_H_5_N_5_O | 1.0463 | HMDB0000132 | C00242 | 14.53±0.67 | 4.58 |
|  | Hypotaurine | 1.23 | 109.0197 | C_2_H_7_NO_2_S | 2.1608 | HMDB0000965 | C00519 | 21.32±1.86 | 8.72 |
|  | Hypoxanthine | 1.34 | 136.0380 | C_5_H_4_N_4_O | 0.0208 | HMDB0000157 | C00262 | 124.01±6.82 | 5.50 |
|  | Indan-1-ol | 10.84 | 134.0727 | C_9_H_10_O | 0.0740 | HMDB0059601 | C01710 | 2870.51±257.20 | 8.96 |
|  | Indole-3-acetate | 4.71 | 117.0574 | C_8_H_7_N | 0.3581 | HMDB0000738 | C00463 | 41.12±5.47 | 13.30 |
|  | Indoleacrylicacid | 4.71 | 187.0626 | C_11_H_9_NO_2_ | 0.2990 | HMDB0000734 | NA | 39.70±3.69 | 9.29 |
|  | Inosine | 1.34 | 268.0797 | C_10_H_12_N_4_O_5_ | 0.7218 | HMDB0000195 | C00294 | 1944.14±282.32 | 14.52 |
|  | Isoquinoline | 4.71 | 129.0575 | C_9_H_7_N | 0.2104 | HMDB0034244 | C06323 | 96.95±5.15 | 5.31 |
|  | Kynurenic acid | 4.86 | 189.0419 | C_10_H_7_NO_3_ | 0.9245 | HMDB0000715 | C01717 | 16.04±2.37 | 14.77 |
|  | LL-Cyclo(leucylprolyl) | 4.29 | 210.1362 | C_11_H_18_N_2_O_2_ | 0.6868 | HMDB0034276 | NA | 1.77±0.24 | 13.29 |
|  | L-Asparticacid | 1.23 | 133.0371 | C_4_H_7_NO_4_ | 0.4597 | HMDB0000191 | C00049 | 1.47±0.18 | 12.52 |
|  | Laurolactam | 11.15 | 197.1773 | C_12_H_23_NO | 1.1167 | NA | NA | 177.53±10.87 | 6.12 |
|  | L-Cystathionine | 1.15 | 222.0668 | C_7_H_14_N_2_O_4_S | 0.4472 | HMDB0000099 | C02291 | 3.28±0.21 | 6.47 |
|  | L-Ergothioneine | 1.29 | 229.0877 | C_9_H_15_N_3_O_2_S | 1.1688 | HMDB0003045 | C05570 | 1.80±0.24 | 13.40 |
|  | Leucine | 2.09 | 131.0942 | C_6_H_13_NO_2_ | 0.5592 | HMDB0000687 | C00123 | 347.75±19.30 | 5.55 |
|  | Leucylproline | 4.36 | 228.1467 | C_11_H_20_N_2_O_3_ | 0.8483 | HMDB0011175 | NA | 5805.72±751.94 | 12.95 |
|  | L-Glutamic acid | 1.23 | 147.0526 | C_5_H_9_NO_4_ | 0.6331 | HMDB0000148 | C00025 | 23.26±3.86 | 16.59 |
|  | L-Histidine | 1.13 | 155.0689 | C_6_H_9_N_3_O_2_ | 0.0351 | HMDB0000177 | C00135 | 3670.45±100.50 | 2.74 |
|  | L-Kynurenine | 3.25 | 208.0841 | C_10_H_12_N_2_O_3_ | 1.3739 | HMDB0000684 | C00328 | 1021.09±102.15 | 10.00 |
|  | L-Lysine | 1.13 | 146.1050 | C_6_H_14_N_2_O_2_ | 0.6405 | HMDB0000182 | C00047 | 158.01±24.15 | 15.29 |
|  | L-Phenylalanine | 3.33 | 165.0784 | C_9_H_11_NO_2_ | 0.4324 | HMDB0000159 | C00079 | 3015.29±181.93 | 6.03 |
|  | L-Pyroglutamic acid | 1.22 | 129.0422 | C_5_H_7_NO_3_ | 0.6353 | HMDB0000267 | C01879 | 10921.79±1342.30 | 12.29 |
|  | L-Serine | 1.22 | 105.0420 | C_3_H_7_NO_3_ | 0.3078 | HMDB0000187 | C00065 | 3674.33±155.43 | 4.23 |
|  | L-Threonine | 1.23 | 87.0318 | C_4_H_9_NO_3_ | 0.0790 | HMDB0000167 | C00188 | 2272.58±117.22 | 5.16 |
|  | Melatonin | 7.28 | 232.1203 | C_13_H_16_N_2_O_2_ | 1.0677 | HMDB0001389 | C01598 | 737.16±37.35 | 5.07 |
|  | Methenolone | 11.94 | 302.2233 | C_20_H_32_O_3_ | 0.9060 | HMDB0041928 | C14778 | 1.52±0.18 | 12.10 |
|  | Methionine | 1.68 | 149.0505 | C_5_H_11_NO_2_S | 0.4320 | HMDB0000696 | C00073 | 11.93±1.91 | 16.03 |
|  | Methyl hexadecanoic acid | 10.57 | 270.2558 | C_17_H_34_O_2_ | 1.8099 | HMDB0061859 | C16995 | 1991.32±171.39 | 8.61 |
|  | Methylimidazoleacetic acid | 1.33 | 140.0583 | C_6_H_8_N_2_O_2_ | 0.5823 | HMDB0002820 | C05828 | 78.76±2.87 | 3.64 |
|  | N6N6N6-Trimethyl-L-lysine | 1.14 | 188.1519 | C_9_H_20_N_2_O_2_ | 0.8174 | HMDB0001325 | C03793 | 1919.60±224.44 | 11.69 |
|  | N6-Acetyl-L-lysine | 1.28 | 188.1154 | C_8_H_16_N_2_O_3_ | 1.1674 | HMDB0000206 | C02727 | 40.86±4.37 | 10.69 |
|  | Nicotinamide | 1.42 | 122.0477 | C_6_H_6_N_2_O | 0.1353 | HMDB0001406 | C00153 | 36.28±2.61 | 7.19 |
|  | N-Methylnicotinamide | 1.22 | 136.0632 | C_7_H_8_N_2_O | 1.5334 | HMDB0003152 | NA | 2036.24±217.92 | 10.70 |
|  | N-Oleoylethanolamine | 14.84 | 325.2968 | C_20_H_35_NO_2_ | 1.3606 | HMDB0002088 | NA | 19.23±1.11 | 5.79 |
|  | Oleamide | 14.47 | 281.2707 | C_18_H_35_NO | 0.7535 | HMDB0002117 | C19670 | 27.03±2.83 | 10.47 |
|  | Ornithine | 1.13 | 132.0896 | C_5_H_12_N_2_O_2_ | 0.6705 | HMDB0000214 | C00077 | 369.27±40.59 | 10.99 |
|  | Glutathione | 1.33 | 307.0838 | C_10_H_17_N_3_O_6_S | 0.9493 | HMDB0000125 | C00051 | 1248.28±83.22 | 6.67 |
|  | Palmitic amide | 14.33 | 255.2550 | C_16_H_33_NO | 0.9479 | HMDB0012273 | NA | 2954.93±184.41 | 6.24 |
|  | Palmitoleic acid | 11.64 | 254.2235 | C_16_H_30_O_2_ | 0.9248 | HMDB0003229 | C08362 | 130.21±7.50 | 5.76 |
|  | Palmitoyl ethanol amide | 13.61 | 299.2812 | C_18_H_37_NO_2_ | 0.6372 | HMDB0002100 | C16512 | 20.57±1.29 | 6.29 |
|  | Palmitoyl carnitine | 11.76 | 399.3334 | C_23_H_45_NO_4_ | 1.4765 | HMDB0000222 | C02990 | 7.69±0.56 | 7.26 |
|  | Phenylacetyl glycine | 6.02 | 193.0733 | C_10_H_11_NO_3_ | 0.0997 | HMDB0000821 | C05598 | 7.25±0.59 | 8.19 |
|  | Pipecolic acid | 1.33 | 129.0787 | C_6_H_11_NO_2_ | 1.0311 | HMDB0000070 | C00408 | 7.19±0.70 | 9.72 |
|  | Prolin amide | 1.13 | 114.0792 | C_5_H_10_N_2_O | 0.1869 | NA | NA | 84.73±14.38 | 16.97 |
|  | Proline | 1.27 | 115.0631 | C_5_H_9_NO_2_ | 0.8502 | HMDB0000162 | C00148 | 113.75±9.33 | 8.20 |
|  | Propionylcarnitine | 2.31 | 217.1307 | C_10_H_19_NO_4_ | 2.3799 | HMDB0000824 | C03017 | 3785.77±169.52 | 4.48 |
|  | Punicic acid | 11.74 | 278.2234 | C_18_H_30_O_2_ | 0.0476 | HMDB0030963 | C08364 | 142.47±23.86 | 16.75 |
|  | Retinal | 12.05 | 284.2129 | C_20_H_28_O | 1.2691 | HMDB0001358 | C00376 | 134.42±11.76 | 8.75 |
|  | Sedanolide | 13.49 | 176.1194 | C_12_H_18_O_2_ | 0.6760 | HMDB0034450 | C17002 | 5.94±0.65 | 11.01 |
|  | Spermidine | 1.06 | 145.1574 | C_7_H_19_N_3_ | 0.2618 | HMDB0001257 | C00315 | 6.03±1.08 | 17.83 |
|  | Sphingosine | 11.07 | 299.2813 | C_18_H_37_NO_2_ | 0.4242 | HMDB0000252 | NA | 39.16±4.54 | 11.59 |
|  | Stearamide | 14.98 | 283.2863 | C_18_H_37_NO | 0.8138 | HMDB0034146 | C13846 | 401.29±54.45 | 13.57 |
|  | Taurine | 1.24 | 125.0144 | C_2_H_7_NO_3_S | 0.9290 | HMDB0000251 | C00245 | 24.91±4.07 | 16.32 |
|  | Taurocholicacid | 7.39 | 515.2900 | C_26_H_45_NO_7_S | 0.8195 | HMDB0000036 | C05122 | 529.89±39.45 | 7.45 |
|  | Tetracosahexaenoic acid | 10.07 | 356.2703 | C_24_H_36_O_2_ | 0.1381 | HMDB0002007 | NA | 15.21±1.65 | 10.83 |
|  | Tetraglyme | 5.24 | 222.1460 | C_10_H_22_O_5_ | 0.6838 | NA | NA | 6.26±0.62 | 9.92 |
|  | Thiamine | 1.18 | 264.1037 | C_12_H_16_N_4_OS | 1.1181 | HMDB0000235 | C00378 | 3.50±0.57 | 16.42 |
|  | Thymine | 2.68 | 126.0426 | C_5_H_6_N_2_O_2_ | 0.8081 | HMDB0000262 | C00178 | 88.40±8.48 | 9.59 |
|  | Tributylphosphate | 11.78 | 266.1636 | C_12_H_27_O_4_P | 0.3925 | NA | NA | 118.99±15.68 | 13.18 |
|  | Tributylamine | 8.00 | 185.2137 | C_12_H_27_N | 0.6013 | NA | NA | 20.88±3.63 | 17.41 |
|  | Tridemorph | 16.26 | 297.3020 | C_19_H_39_NO | 0.6027 | HMDB0031810 | C11285 | 5.72±0.24 | 4.18 |
|  | Trigonelline | 1.27 | 137.0472 | C_7_H_7_NO_2_ | 0.5009 | HMDB0000875 | C01004 | 65.57±6.31 | 9.62 |
|  | Umbelliferone | 9.11 | 162.0311 | C_9_H_6_O_3_ | 0.5439 | HMDB0029865 | C09315 | 86.24±14.44 | 16.74 |
|  | Uracil | 1.35 | 112.0272 | C_4_H_4_N_2_O_2_ | 1.0356 | HMDB0000300 | C00106 | 426.58±39.78 | 9.32 |
|  | Uricacid | 1.68 | 168.0277 | C_5_H_4_N_4_O_3_ | 0.3388 | HMDB0000289 | C00366 | 1.40±0.16 | 11.15 |
|  | Valine | 1.70 | 117.0788 | C_5_H_11_NO_2_ | 0.1652 | HMDB0000883 | C00183 | 1264.75±119.28 | 9.43 |
|  | Vanillin | 6.37 | 152.0469 | C_8_H_8_O_3_ | 0.6170 | HMDB0012308 | C00755 | 4.58±0.42 | 9.18 |
|  | *α-*Aminocaprylicacid | 5.96 | 159.1254 | C_8_H_17_NO_2_ | 1.2014 | HMDB0000991 | NA | 3.47±0.36 | 10.46 |
|  | *α-*Linolenoylethanolamide | 13.43 | 321.2654 | C_20_H_39_NO_2_ | 0.4668 | HMDB0013624 | C13828 | 16.41±1.61 | 9.79 |
|  | *β*-Cortolone | 8.32 | 348.2289 | C_10_H_14_N_5_O_7_P | 2.2887 | HMDB0013221 | C05481 | 7.26±0.72 | 9.86 |

*NA means not available

Table S2. List of the 52 metabolites which were screened by one-way analysis of variance of metabolites in control group, DSS group, CA group and GA group.

| NO. | Name | t_R_(min) | HMDB | KEGG | VIP>1 | Relative intensity | | | |
| --- | --- | --- | --- | --- | --- | --- | --- | --- | --- |
|  |  |  |  |  |  | Control | DSS | CA | GA |
|  | Propionyl carnitine | 2.31 | HMDB0000824 | C03017 | ∆^a^ | 384.21±98.64 | 5700.72±2028.84^c^ | 588.66±375.78 | 847.76±530.07 |
|  | 5'-Methylthioadenosine | 4.30 | HMDB0001173 | C00170 | ∆ | 1098.98±412.73 | 177.72±98.85^c^ | 332.70±173.15^e^ | 144.91±32.16^g^ |
|  | Arachidonic acid | 14.09 | HMDB0001043 | C00219 | ∆ | 5997.26±1389.78 | 2527.74±396.91^c^ | 3919.27±744.50 ^e^ | 4579.15±951.61 |
|  | Asymmetric dimethylarginine | 1.22 | HMDB0001539 | C03626 | ∆ | 1460.57±176.09 | 3332.94±550.11^c^ | 2628.13±663.53^e^ | 2836.92±632.09^g^ |
|  | 2-Hydroxycinnamic acid | 1.80 | HMDB0002641 | C01772 | ∆ | 41814.40±4160.80 | 46614.17±2664.52^b^ | 43477.82±3774.49 | 45783.97±4025.79^f^ |
|  | Eicosapentaenoic acid | 12.05 | HMDB0001999 | C06428 | ∆ | 598.51±157.37 | 1678.50±496.18^c^ | 1621.33±624.38^e^ | 1823.51±580.45^g^ |
|  | Hypotaurine | 1.23 | HMDB0000965 | C00519 | ∆ | 284.55±70.53 | 4235.41±2659.97^c^ | 358.70±289.65 | 606.52±428.58 ^f^ |
|  | 7-Methylguanosine | 2.10 | HMDB0001107 | NA | ∆ | 38.09±6.43 | 30.11±10.26^b^ | 69.08±28.06 | 90.16±21.60^f^ |
|  | L-Glutamic acid | 1.23 | HMDB0000148 | C00025 | - | 43708.36±3309.61 | 32873.93±4521.91^c^ | 49026.16±6615.43 | 45206.38±8915.42 |
|  | Retinal | 12.05 | HMDB0001358 | C00376 | ∆ | 22.37±7.07 | 64.89±18.81^c^ | 64.41±27.57^d^ | 71.68±23.30^f^ |
|  | D-Maltose | 1.21 | HMDB0000163 | C00208 | ∆ | 1779.42±483.21 | 1155.87±283.50^b^ | 1747.31±317.29 | 1807.18±343.14 |
|  | 3-Hydroxypicolinic acid | 1.99 | HMDB0013188 | C18620 | - | 491.67±158.22 | 92.85±40.66^c^ | 302.80±154.85^d^ | 210.48±85.69^f^ |
|  | L-Aspartic acid | 1.23 | HMDB0000191 | C00049 | - | 1554.21±229.08 | 1567.27±281.41^b^ | 2305.17±653.31^d^ | 2752.15±634.34^g^ |
|  | 4-Pyridoxic acid | 2.02 | HMDB0000017 | C00847 | ∆ | 144.45±66.08 | 65.88±29.11^b^ | 131.24±19.31 | 164.84±46.90 |
|  | L-Pyroglutamic acid | 1.22 | HMDB0000267 | C01879 | - | 54674.52±10108.24 | 40393.45±5735.67^c^ | 39412.07±7405.37^e^ | 32386.24±5305.82^g^ |
|  | 2-Ethylamino-1-phenylcutane | 10.86 | NA* | NA | - | 2045.60±449.19 | 1318.40±406.96^c^ | 1377.93±208.93^e^ | 1087.82±196.00^g^ |
|  | Cortisol | 8.62 | HMDB0000063 | C00735 | ∆ | 11.69±9.99 | 506.20±352.53^c^ | 21.59±20.54 | 73.07±52.63 ^g^ |
|  | Guanine | 1.34 | HMDB0000132 | C00242 | ∆ | 187.17±47.36 | 336.74±109.82^c^ | 245.23±58.58 | 263.57±57.07^f^ |
|  | Glucose 1-phosphate | 1.26 | HMDB0001586 | C00103 | ∆ | 578.68±137.07 | 235.28±78.19^c^ | 400.70±75.89^e^ | 322.36±76.24^g^ |
|  | Uracil | 1.35 | HMDB0000300 | C00106 | ∆ | 4359.67±764.58 | 2963.04±608.96^b^ | 5243.61±1162.11 | 3965.74±1520.60 |
|  | L-Carnitine | 1.24 | HMDB0000062 | C00318 | ∆ | 17807.48±3512.00 | 38793.25±6811.27^c^ | 20469.79±5089.24 | 23672.54±7816.16 |
|  | Docosapentaenoic acid | 14.22 | HMDB0006528 | C16513 | ∆ | 444.91±91.67 | 166.56±53.25^c^ | 195.88±60.57^e^ | 184.92±67.95^g^ |
|  | Adenosine | 1.70 | HMDB0000050 | C00212 | ∆ | 730.08±120.65 | 250.33±64.90^c^ | 357.95±117.34^e^ | 287.89±137.51^g^ |
|  | β-Cortolone | 8.32 | HMDB0013221 | C05481 | ∆ | 18.50±12.94 | 234.38±106.55^c^ | 5.97±4.67 | 28.75±24.15 ^f^ |
|  | Corticosterone | 8.08 | HMDB0001547 | C02140 | ∆ | 3.72±2.15 | 73.06±37.02^c^ | 2.27±0.84 | 37.94±30.86 |
|  | Methenolone | 11.94 | HMDB0041928 | C14778 | - | 64.65±15.56 | 115.30±21.72^c^ | 136.57±50.60^d^ | 169.54±45.97^g^ |
|  | Andrographolide | 9.62 | NA | NA | ∆ | 2.31±0.62 | 42.36±22.28^c^ | 4.05±3.04 | 8.07±5.67 |
|  | 6-Methylquinoline | 5.22 | HMDB0033115 | NA | - | 5.77±1.38 | 22.75±19.17^b^ | 120.10±21.40^d^ | 130.42±30.00^f^ |
|  | Glycylproline | 1.70 | HMDB0000721 | NA | ∆ | 116.59±19.36 | 213.04±45.74^c^ | 164.62±29.84^d^ | 154.76±49.39 |
|  | Thiamine | 1.18 | HMDB0000235 | C00378 | ∆ | 1213.24±172.57 | 721.46±104.90^c^ | 1244.25±330.44 | 936.37±77.03 ^g^ |
|  | L-Lysine | 1.13 | HMDB0000182 | C00047 | - | 29151.52±2521.68 | 33767.78±3753.10^c^ | 33830.65±2981.41^e^ | 33761.14±2650.19^f^ |
|  | Methyl palmitate | 10.57 | HMDB0061859 | C16995 | ∆ | 1232.15±479.27 | 991.14±697.34^b^ | 1958.65±485.88^e^ | 1765.57±524.24 |
|  | 1-Linoleoyl-glycerol | 11.37 | HMDB0011568 | NA | - | 97.03±38.75 | 25.16±7.68^c^ | 45.01±26.57 | 40.44±14.31^g^ |
|  | Glutathione | 1.33 | HMDB0000125 | C00051 | - | 39617.25±6036.10 | 22513.56±4980.63^c^ | 34831.89±6374.22 | 34240.51±8982.61 |
|  | N6-Acetyl-L-lysine | 1.28 | HMDB0000206 | C02727 | ∆ | 265.83±37.06 | 507.29±141.26^c^ | 309.20±95.39 | 350.36±83.69 ^f^ |
|  | 1-Methylhistidine | 1.21 | HMDB0000001 | C01152 | ∆ | 24.66±5.94 | 161.44±147.60^c^ | 57.68±37.67 | 46.83±24.04^g^ |
|  | Proline | 1.27 | HMDB0000162 | C00148 | - | 33937.34±3695.64 | 44781.44±2913.57^c^ | 44552.87±6466.65^d^ | 46503.85±10299.32^g^ |
|  | N-Methylnicotinamide | 1.22 | HMDB0003152 | NA | ∆ | 72.84±27.93 | 294.83±175.09^c^ | 235.06±174.69^d^ | 173.92±75.76^f^ |
|  | Laurolactam | 11.15 | NA | NA | ∆ | 37.00±15.58 | 25.87±4.44^b^ | 26.54±5.97 | 23.79±7.61^f^ |
|  | L-Tryptophan | 4.71 | HMDB0013609 | C00525 | - | 19095.86±3431.75 | 19538.41±1573.71^b^ | 21380.54±1237.99 | 23131.40±1787.06^f^ |
|  | 5-Hydroxyindole-3-acetic acid | 3.26 | HMDB0000763 | C05635 | ∆ | 803.38±155.86 | 2930.29±1433.09^c^ | 1543.38±1302.91 | 2375.03±1305.74^f^ |
|  | Cytosine | 1.28 | HMDB0000630 | C00380 | - | 1262.22±207.41 | 1102.20±154.74^b^ | 1093.37±293.15 | 1169.39±263.20 |
|  | L-Serine | 1.22 | HMDB0000187 | C00065 | - | 33888.95±6847.63 | 26077.73±3462.08^c^ | 23371.27±5026.17^e^ | 19459.48±3664.87^g^ |
|  | Cytidine | 1.28 | HMDB0000089 | C00475 | - | 906.25±203.81 | 632.67±155.92^b^ | 714.93±241.36 | 717.68±183.08 |
|  | L-Kynurenine | 3.25 | HMDB0000684 | C00328 | ∆ | 654.22±129.20 | 2302.36±1173.26^c^ | 1233.12±1104.86 | 1653.55±699.91^g^ |
|  | Benzotriazole | 5.80 | NA | NA | - | 115.32±30.24 | 117.72±40.84^b^ | 60.61±32.43^e^ | 106.96±27.18 |
|  | Adenosine 5'-monophosphate | 4.52 | HMDB0000045 | C00020 | - | 454.96±129.09 | 307.76±56.43^b^ | 503.40±81.84 | 440.64±38.22 |
|  | 2-Hydroxypropyl methacrylate | 6.68 | NA | NA | - | 13422.12±3602.44 | 10060.25±2172.58^c^ | 11174.65±1769.03 | 8035.20±2318.02^g^ |
|  | Palmitoyl carnitine | 11.76 | HMDB0000222 | C02990 | ∆ | 39.64±15.63 | 131.29±32.20^c^ | 39.09±24.66 | 87.65±56.63 |
|  | Palmitoleic acid | 11.64 | HMDB0003229 | C08362 | - | 170.86±37.15 | 254.46±62.47^c^ | 218.10±32.63^e^ | 195.55±44.31 |
|  | Indole-3-acetate | 4.71 | HMDB0000734 | NA | - | 19054.46±3424.91 | 19968.82±1833.57^b^ | 20716.54±2195.55 ^e^ | 23069.60±1769.34^f^ |
|  | Cortisone | 8.38 | HMDB0002802 | C00762 | ∆ | 1.81±0.46 | 33.18±27.09^b^ | 2.02±0.92 | 10.02±8.24 ^f^ |

*NA means not available. ^a^ ∆ means VIP>1 in OPLS-DA. ^b^DSS group compared with control group, *p*<0.05. ^c^DSS group compared with control group, *p*<0.01. ^d^CA group compared with control group, *p*<0.05. ^e^CA group compared with control group, *p*<0.01. ^f^GA group compared with control group, *p*<0.05. ^g^GA group compared with control group, *p*<0.01.
